# Bone Compatibility of Experimental Ti–Ag and Ti–Cu Alloy Dental Implants in a Beagle Dog Model

**DOI:** 10.3390/jfb17040198

**Published:** 2026-04-18

**Authors:** Yasumitsu Ohtsuka, Taichi Tenkumo, Masatoshi Takahashi, Yasuhiro Nakanishi, Hiroaki Takebe, Takashi Nezu

**Affiliations:** 1 Ohtsuka Dental Clinic, 77-18 Miyukigahara-machi, Utsunomiya 321-0982, Japan; info@ohtsuka-dental.com; 2 Division of Advanced Prosthetic Dentistry, Tohoku University Graduate School of Dentistry, 4-1 Seiryo-machi, Aoba-ku, Sendai 980-8575, Japan; taichi.tenkumo.a3@tohoku.ac.jp; 3 Division of Biomaterials and Bioengineering, School of Dentistry, Health Sciences University of Hokkaido, 1757 Kanazawa, Tobetsu-cho, Hokkaido 061-0293, Japan; tnezu@hoku-iryo-u.ac.jp; 4 Division of Fixed Prosthodontics and Oral Implantology, School of Dentistry, Health Sciences University of Hokkaido, 1757 Kanazawa, Tobetsu-cho, Hokkaido 061-0293, Japan; nakanisi@hoku-iryo-u.ac.jp; 5 Division of Histology, Health Sciences University of Hokkaido, 1757 Kanazawa, Tobetsu-cho, Hokkaido 061-0293, Japan; takebeh@hoku-iryo-u.ac.jp

**Keywords:** titanium alloys, dental implants, biocompatibility, Ti–Ag alloys, Ti–Cu alloys

## Abstract

Titanium–silver (Ti–Ag) and titanium–copper (Ti–Cu) alloys have been developed to improve the mechanical properties and machinability of titanium (Ti) for dental applications while maintaining corrosion resistance comparable to that of pure Ti. Herein, cylindrical dental implants composed of experimental Ti–20Ag, Ti–30Ag, Ti–5Cu, and Ti–10Cu (mass%) alloys were fabricated and implanted into the jawbones of beagle dogs to evaluate bone compatibility. Pure Ti and Ti–6Al–4V alloy implants were used as controls. Because the implant surfaces were mechanically polished, the experimental alloys, which exhibited higher hardness than Ti, showed lower surface roughness than Ti. Radiographic observations revealed no remarkable bone resorption around any implants during the experimental period. Histological evaluation demonstrated new bone formation and partial bone contact around implants at 1 and 3 months post-implantation. Although the bone–implant contact ratio was relatively low owing to the cylindrical implant design and limited initial stability, no significant differences were observed between the experimental alloys and Ti. These results indicate that Ti–Ag and Ti–Cu alloys improve mechanical properties while maintaining bone compatibility comparable to that of Ti, suggesting their potential as candidate materials for dental implant applications, particularly for narrow dental implants.

## 1. Introduction

Various materials, including cobalt–chromium alloys and sapphire, have been used during the early development of dental implants. Since the discovery of direct bone-bonding of titanium (Ti), known as osseointegration, by Per-Ingvar Brånemark, Ti has become the standard material for dental implants [1,2].

In recent years, the demand for narrow, short implants has increased to accommodate patients with limited bone volume and enable less invasive treatments [3,4]. However, the mechanical strength of commercially pure Ti is sometimes insufficient, and clinical problems, such as fixture fractures due to inadequate strength, have been reported [5]. To overcome this limitation, high-strength Ti alloys such as Ti–6Al–4V ELI and Ti–6Al–7Nb are currently used [6]. However, these alloys contained aluminum (Al) and vanadium (V): Al is associated with neurotoxicity and Alzheimer’s disease, and V exhibits relatively strong cytotoxicity [7,8]. Although the amounts released are extremely small, the possible release of these metal ions during long-term clinical use has raised concerns regarding their biological effects.

In recent years, Ti-based alloys have been developed to achieve a lower elastic modulus by adding non-toxic β-phase-stabilizing elements (such as Nb, Zr, Ta, and Mo) to titanium [9,10]. In our previous studies, we have also developed various Ti alloys to improve mechanical properties and investigated their characteristics [11,12,13,14,15]. Among them, Ti–Ag and Ti–Cu alloys exhibit particularly favorable mechanical properties. The strength of Ti–Ag alloys increases with Ag content, reaching >1.6 times that of Ti at 20 mass% Ag [11]. Similarly, the strength of Ti–Cu alloys increased with Cu addition and was approximately twice that of Ti at 5 mass% Cu [11]. Both alloys retained sufficient ductility and corresponded to type 4 metallic materials as per the ISO classifications for dental metals. Furthermore, Ti is generally regarded as a difficult-to-machine material; however, these alloys exhibit improved machinability in cutting and grinding processes [16,17]. As dental implants require precise machining to achieve accurate connections with abutments, alloys with superior machinability are considered advantageous. Corrosion resistance is an essential property of materials used in oral environments. Electrochemical corrosion tests and ion release analyses demonstrated that these alloys exhibit a corrosion resistance comparable to that of Ti [18,19]. Even if small amounts of Ag or Cu ions are released, these elements are generally considered to exhibit good biocompatibility and a low incidence of allergic reactions [20,21].

Among these alloys, Ti–Ag alloys have been extensively investigated. Immersion tests in simulated body fluids have shown that calcium phosphate spontaneously precipitates on Ti–Ag alloy surfaces, as it does on Ti [22]. This property is advantageous for implant applications. Another notable characteristic of Ti–Ag alloys is their ability to suppress biofilm formation. When biofilms of *Streptococcus mutans* and *Streptococcus sobrinus* were formed on alloy surfaces, the amount of biofilm formed on Ti–Ag alloys was lower than that on Ti, suggesting the inhibition of biofilm formation [23]. Importantly, Ti–Ag alloys exhibit good corrosion resistance and do not release Ag ions; therefore, this effect is not attributable to bactericidal activity caused by Ag ion release. Thus, this antibiofilm effect is biologically safe [23]. Such properties may be beneficial for implant abutments that penetrate the mucosa and connect the body’s internal and external environments and may also help suppress the progression of peri-implantitis.

In contrast, the antibiofilm properties of Ti–Cu alloys remain unclear. Like Ag, Cu, which has long been used in dentistry, exhibits antibacterial activity via the release of its ions. However, results obtained from Ti–Ag–Cu alloys, in which Cu is added as a third element, suggest that Cu has a relatively limited ability to confer anti-biofilm properties to Ti [13]. Nevertheless, Ti–Cu alloys with up to 5 mass% Cu exhibit corrosion resistance comparable to that of Ti [19], and their favorable mechanical properties and machinability [11,16] make them suitable for implant materials. Therefore, it is important to investigate their biological behavior when used in dental implants.

In the present study, cylindrical dental implants made of Ti–Ag and Ti–Cu alloys were fabricated and implanted into the jawbones of beagle dogs. Based on previous evaluations of the mechanical properties, machinability, and corrosion resistance, Ti–20Ag and Ti–5Cu were representative compositions. Furthermore, Ti–30Ag and Ti–10Cu were investigated to examine the effects of increased alloying content, which may promote precipitation of intermetallic compounds such as Ti_2_Ag and Ti_2_Cu. Finally, histological observations and bone–implant contact (BIC) measurements were performed to evaluate the bone compatibility of the experimental alloys. Our study hypothesis was that the developed Ti–Ag and Ti–Cu alloys do not significantly compromise the bone biocompatibility of titanium.

## 2. Materials and Methods

### 2.1. Implant Fabrication

The compositions of the experimental Ti–Ag and Ti–Cu alloys were Ti–20Ag, Ti–30Ag, Ti–5Cu, and Ti–10Cu (mass%). Ti sponge (>99.8%; grade S-90; Osaka Titanium Technologies, Amagasaki, Japan), silver (>99.99%; Hirano Seizaemon Shoten, Tokyo, Japan), and copper (>99.99%; Research Institute for Electric and Magnetic Materials, Sendai, Japan) were weighed to obtain the desired alloy compositions. The weighed elements were melted in an argon arc melting furnace (TAM-4S, Tachibana Riko, Sendai, Japan) under a high-purity argon atmosphere (>99.9999% purity). The ingots were melted six times, and the button was flipped between each melting cycle to ensure chemical homogeneity. Button-shaped Ti-alloy ingots (approximately 30 g) were produced. Pure Ti ingots were prepared using the same procedure.

The ingots were cast into cylindrical rods (5 mm diameter and 30 mm length) using a dental Ti casting machine (Autocast HC-III, GC Corp., Tokyo, Japan). The cast rods were subsequently machined by a professional medical Ti-processing company (T and I Co., Ltd., Saitama, Japan) to produce cylindrical implants with a diameter of 3.25 mm (tolerance: −0.05 mm) and a length of 8 mm. In addition, cylindrical implants of the same dimensions were fabricated from medical-grade Ti–6Al–4V ELI alloy rods (4 mm diameter) for use as controls. The surfaces of all implants were mechanically polished.

### 2.2. Hardness and Surface Roughness

The bulk hardness of the implants was measured using a micro-Vickers hardness tester (HM-221; Mitutoyo, Kawasaki, Japan) under a load of 1.961 N and a dwell time of 15 s (*n* = 10).

The surface roughness was measured using a surface profilometer (Surfcom 480A, Tokyo Seimitsu, Tokyo, Japan) (*n* = 10). The measurements were performed along the longitudinal axis of the cylindrical implant surface. The measurement conditions were an evaluation length of 4.0 mm, a stylus speed of 0.6 mm/s, and a cutoff value of 0.8 mm. The surface roughness was characterized using the height parameter Ra.

### 2.3. Implantation Experiment

#### 2.3.1. Experimental Animals

Six male beagles (18 months old; Institute for Animal Reproduction, Kasumigaura, Japan) were used in this study. All procedures were performed in accordance with the principles of laboratory animal care and relevant national regulations. The study protocol was approved by the Institutional Animal Care and Use Committee of Tohoku University (Permit No. 2012DnA-131).

#### 2.3.2. Implant Placement Procedure

All surgical procedures were performed under general anesthesia induced by intramuscular injection of medetomidine hydrochloride (Domitor^®^, Nippon Zenyaku Kogyo Co., Ltd., Koriyama, Japan; 0.1 mL/kg) and ketamine hydrochloride (Ketalar^®^, Daiichi Sankyo Co., Ltd., Tokyo, Japan; 0.1 mL/kg). Local anesthesia was achieved using 2% lidocaine containing epinephrine (1:80,000) (Xylocaine^®^, AstraZeneca, Osaka, Japan).

**Figure 1:**
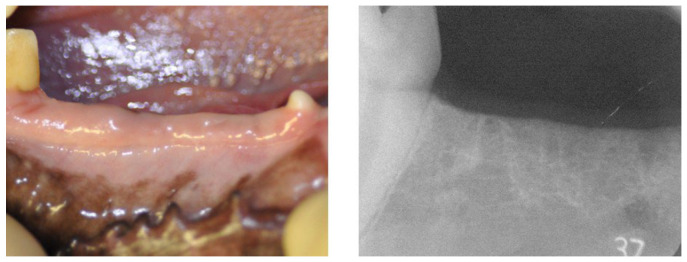
Gross view and radiographs of the canine mandible at 3 months after tooth extraction, showing the healed extraction site prior to implant placement.

The mandibular second, third, and fourth premolars on both sides were extracted, and a healing period of three months was allowed. Healing of the extraction sites and recovery of the alveolar bone were confirmed by visual inspection and radiographic examination prior to implant placement (Figure 1).

**Figure 2:**
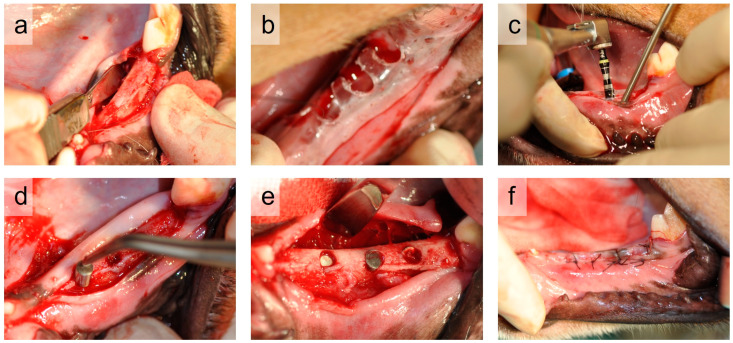
Representative procedure for implant placement in the canine mandible: (**a**) elevation of the mucoperiosteal flap; (**b**) determination of implant position; (**c**) implant osteotomy; (**d**) implant insertion; (**e**) placement of three implants per side; and (**f**) wound closure with primary intention.

During the implantation surgery, a mucosal incision was made on the buccal side of the alveolar ridge, and full-thickness buccal and lingual mucoperiosteal flaps were elevated (Figure 2a). Implant placement sites were determined using a custom-made surgical guide prepared in advance (Figure 2b). Implant osteotomies were performed with copious saline irrigation using a dental implant motor (Implant Motor IM-II; GC Corp., Tokyo, Japan). A round bur and sequential drills were used, and the final osteotomy was performed using a 3.25-mm twist drill to a depth of 8 mm from the crestal bone level (Figure 2c). The sterilized implant bodies were then inserted into the prepared osteotomies (Figure 2d). Six implants were placed on each animal, three on each side of the mandible, with one implant of each of the six types (Figure 2e). A minimum distance of 3 mm was maintained between adjacent implants or between the implants and natural teeth. To minimize positional bias, the placement locations of the implant types were alternated among animals such that each animal received all implant types at different positions. After implantation, the flaps were sutured for complete wound closure using absorbable sutures (Vicryl^®^, 4-0, Johnson & Johnson, New Brunswick, NJ, USA) (Figure 2f). Atipamezole hydrochloride (Antisedan^®^, Nippon Zenyaku Kogyo Co., Ltd., Koriyama, Japan; 0.3 mL) was administered intramuscularly to reverse anesthesia.

Postoperatively, cefcapene pivoxil hydrochloride hydrate (15 mg/kg; Flomox Tablets 75 mg; Shionogi & Co., Ltd., Osaka, Japan) was administered orally for three days to prevent infection. The animals were fed a soft diet for two weeks after surgery.

### 2.4. Clinical and Radiographic Evaluation

Clinical examinations and intraoral radiographs were performed under general anesthesia at the time of implantation and at 1, 2, and 3 months post-surgery to evaluate signs of inflammation around the implants and peri-implant bone resorption. Radiographs were obtained using a dental X-ray unit (MAX-DC70; Morita Corp., Osaka, Japan) with an instant dental film (ISO size 4A; Hanshin Technical Laboratory Ltd., Nishinomiya, Japan) under standardized conditions (60 kV, 10 mA, 0.5 s). A custom positioning jig was used to ensure standardized radiographic positioning [24].

### 2.5. Histology and BIC Measurement

The implantation periods were 1 month (three animals) and 3 months (three animals), determined based on previous similar animal studies evaluating bone healing [25,26]. After each observation period, the animals were euthanized under general anesthesia by intramuscular administration of sodium pentobarbital (Nembutal^®^, Dainippon Sumitomo Pharma Co., Ltd., Osaka, Japan; 0.5 mL/kg), followed by perfusion fixation with 10% neutral buffered formalin (pH 7.4). The mandibular bone containing the implants was excised and immersed in the same fixative for 1 week.

After fixation, the specimens were carefully sectioned in the buccolingual direction between the implants using a micro-cutting machine (BS-300CP, Meiwa Fosis Co., Ltd., Tokyo, Japan) to avoid damage to the peri-implant bone. The specimens were then dehydrated and embedded in methyl methacrylate (MMA) resin, according to standard procedures. Sections were cut in the mesiodistal direction near the central region between the buccal and lingual sides of each implant, and ground sections approximately 20 μm thick were prepared. Sections were stained with hematoxylin and eosin (H&E) and examined by light microscopy.

Bone–implant contact (BIC) was defined as the percentage of the implant surface in direct contact with bone relative to the total implant surface length within the bone. BIC was calculated for the entire implant surface located within the bone using ImageJ software (version 1.54p; National Institutes of Health, Bethesda, MD, USA). No data were excluded, and all samples were analyzed (*n* = 3). Statistical analysis was performed using nonparametric tests.

### 2.6. Statistical Analysis

Hardness and surface roughness data were analyzed using one-way analysis of variance (ANOVA), followed by Tukey’s post hoc test. BIC differences were analyzed using the Kruskal–Wallis test. When a significant difference was detected, pairwise comparisons were performed using Bonferroni correction. The significance level was set at α = 0.05.

## 3. Results

### 3.1. Hardness and Surface Roughness

**Figure 3:**
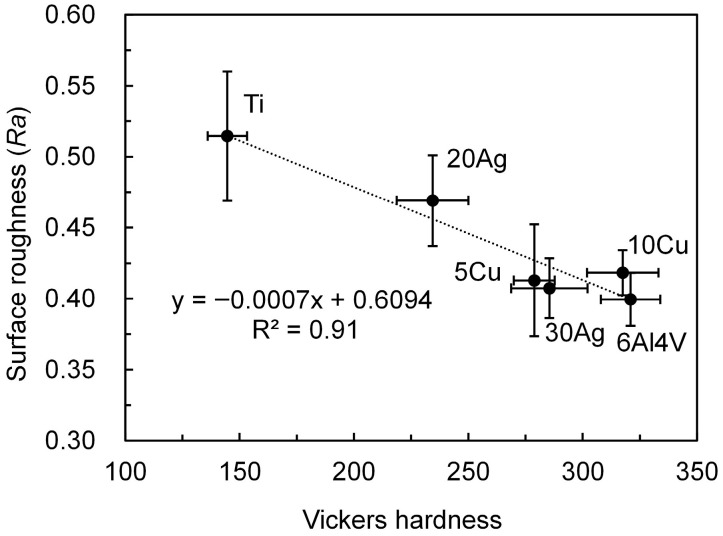
Hardness and surface roughness of the implants.

The hardness and surface roughness of the implants are illustrated in Figure 3. All Ti alloys exhibited significantly higher hardness than pure Ti (~145 HV, *p* < 0.01). Among the alloys, Ti–20%Ag showed the lowest hardness (~235 HV), which was significantly lower than that of the other alloys (>275 HV, *p* < 0.01). Surface roughness was significantly lower for all Ti alloys compared with Ti (0.51 µm, *p* < 0.01). Ti–20%Ag had slightly higher Ra (0.47 µm) than the other alloys (<0.42 µm, *p* < 0.01). The hardness and surface roughness were strongly and negatively correlated (R^2^ = 0.9).

### 3.2. Clinical Observation and Radiographs

During the experimental period, no implant exposure, implant loss, or inflammatory findings suggestive of infection, such as suppuration or bleeding, were observed around the implant sites in any group.

**Figure 4:**
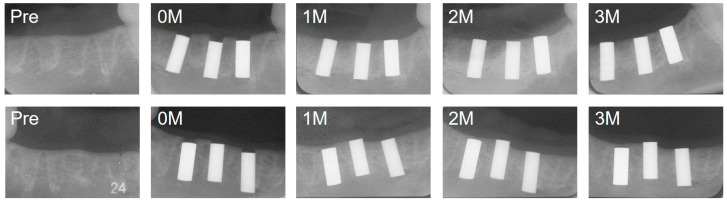
Serial radiographs of implant placement sites. Images were obtained before implantation (Pre), immediately after implantation (0M), and at 1, 2, and 3 months post-implantation. Upper row (left to right): Ti, 5Cu, and 20Ag; lower row (left to right): 30Ag, 10Cu, and 6Al4V.

Representative intraoral radiographs taken at implantation and 1, 2, and 3 months post-implantation are shown in Figure 4. In both pure Ti and experimental Ti alloy implants, slight crestal bone remodeling was observed over time, resulting in a cortical bone level corresponding to the upper margin of the implants. No marked radiolucent or radiopaque changes were observed around the lateral surfaces or the apical regions of the implants during the observation period, significant peri-implant bone resorption, or any apparent influence of the implant position.

### 3.3. Histology

**Figure 5:**
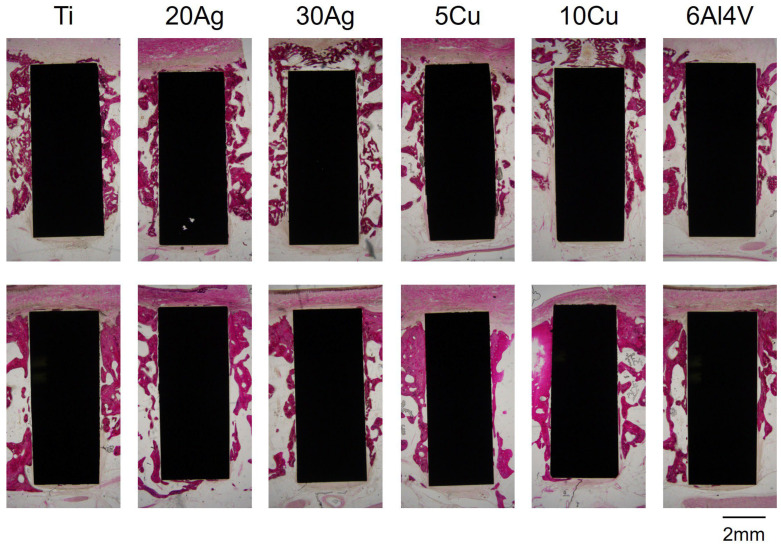
Hematoxylin and eosin (H&E) stained sections. Upper row: 1 month; lower row: 3 months after implantation.

**Figure 6:**
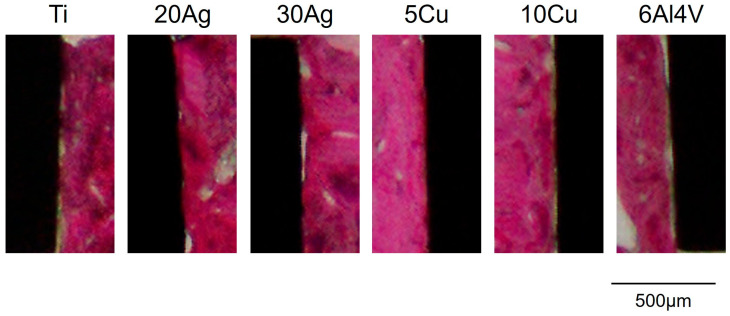
Higher-magnification H&E-stained image showing direct bone-to-implant contact at 3 months after implantation.

Representative H&E-stained sections are shown in Figure 5 and Figure 6. Figure 5 shows an overall view of the implants and surrounding bone, while Figure 6 provides a higher-magnification image highlighting areas of direct bone-to-implant contact. At 1 and 3 months post-implantation, the implants in all groups were surrounded by bone and fibrous tissue. No infiltration of inflammatory cells, such as neutrophils or lymphocytes, was observed. The thickness of the bone surrounding the implants tended to increase between 1 and 3 months post-implantation. Bone attachment to the implant surfaces also tended to increase at 3 months compared with 1 month for all implant types. Small gaps between the bone and the implant surfaces were occasionally observed.

### 3.4. BIC

**Figure 7:**
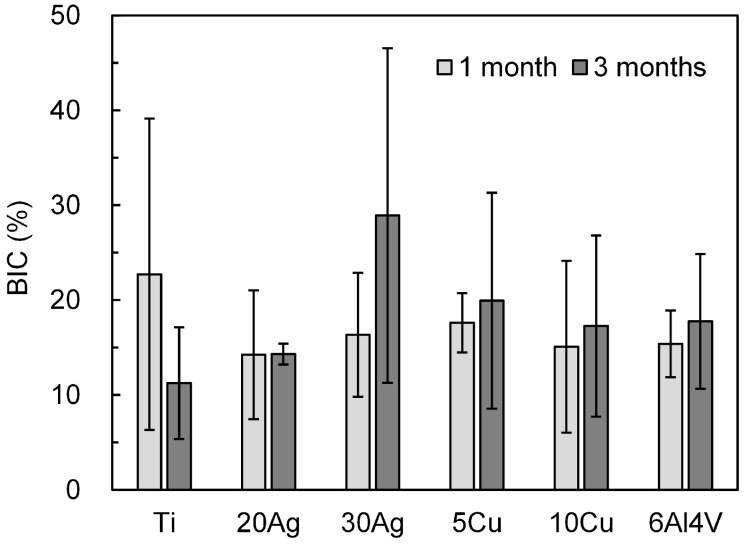
Bone–implant contact ratio (BIC).

The BIC values calculated from the histological sections are shown in Figure 7. No significant differences in BIC were observed between the groups (pure Ti and experimental Ti alloys) or between the observation periods (1 and 3 months) (*p* > 0.05), and relatively large standard deviations were noted. The BIC value of pure Ti was approximately 23% at 1 month and slightly lower at 3 months. After 1 month, the experimental Ti alloys exhibited slightly lower BIC values than those of pure Ti. In contrast, the BIC values of all the experimental Ti alloys tended to increase at 3 months compared with 1 month. The BIC values of the Ti–Ag, Ti–Cu, and Ti-6Al-4V alloys were comparable.

## 4. Discussion

### 4.1. Implant Fabrication

In this study, cylindrical implants were fabricated by first producing alloy ingots by casting and then machining them into rods. Ti and its alloys are susceptible to contamination during casting. For example, the intrusion of air into an argon atmosphere during melting, or excessive reactions with the investment material due to overheating, may cause impurities such as oxygen, carbon, and silicon to dissolve into the metal, thereby significantly increasing hardness. Despite the different investment materials and casting systems used in this study compared with those in previous reports, the hardness values of Ti and Ti alloys were consistent with previously reported data [11,12]. This finding suggests that the cast materials were not significantly contaminated by impurities and their mechanical properties, such as strength and elongation, were comparable to those previously reported. Furthermore, it is unlikely that the functional properties of Ti–Ag alloys, such as inhibition of biofilm formation and spontaneous calcium phosphate deposition, were impaired.

The cast rods were then machined into cylindrical implants. The surface roughness of the fabricated implants was negatively correlated with hardness. Generally, it has been reported that machining behavior changes with increasing material hardness, which may result in reduced surface roughness [22]. Although the surface roughness of implants should be standardized, it is difficult to achieve identical surface roughness when machining materials with different hardnesses. One possible approach is mirror polishing; however, most commercially available dental implants have roughened surfaces resulting from treatments such as sandblasting or alkali etching [2]. Therefore, mirror-polished surfaces do not reflect the clinically relevant conditions. Previous studies have reported that moderately rough surfaces are advantageous for bone integration [2,27]. In this study, pure Ti exhibited the highest surface roughness, whereas the experimental Ti alloy had a significantly lower surface roughness. Therefore, the experimental alloys were evaluated under relatively unfavorable conditions for bone integration. If comparable results are obtained under such conditions, the experimental alloys are promising materials for dental implants.

### 4.2. Implant Placement Surgery in Beagle Dogs

Ti is classified as a bioinert material that can achieve osseointegration, in which the implant surface is in direct contact with bone tissue without intervening soft tissue. In contrast, cobalt–chromium alloys used in artificial joints and stainless steel used in fracture fixation plates normally form a fibrous connective tissue layer at the bone–implant interface. Ti implants may not achieve osseointegration if an infection occurs during or immediately following placement, resulting in fibrous encapsulation and early implant failure [28]. Herein, no macroscopic abnormalities, such as implant loss or infection of the surrounding tissues, were observed during the observation period. Radiographic examinations revealed no marked alveolar bone resorption around the implant. These findings indicate that the surgical procedures were performed without major complications.

### 4.3. Histological Evaluation

Histological observations of H&E-stained sections revealed a small gap between the implant surface and the bone in all samples, including pure Ti. This gap may be partly attributed to artifacts arising during specimen preparation, such as detachment caused by polymerization shrinkage of the embedding resin or mechanical stresses during sectioning and polishing. However, these factors alone cannot fully account for the observed gaps.

Another possible explanation is the presence of a gap at the time of implant placement and insufficient primary stability. Excessive bone compression during implant placement may increase initial BIC, although it carries the risk of bone damage [29,30,31]. Since the primary objective of this study was to evaluate the biological affinity between bone and experimental alloys, the implants were fabricated with a slightly negative tolerance relative to the drill diameter to minimize mechanical damage to the bone. Thus, a small gap inevitably formed between the implant and the osteotomy site at the time of placement. Moreover, slight deviations during drilling may increase the gap. Although osseointegration can occur with small gaps, sufficient primary stability is required [32]. Compared to screw-type implants, cylindrical implants provide weaker primary stability. Insufficient primary stability may induce micromotion at the bone–implant interface, potentially leading to fibrous encapsulation instead of osseointegration [33,34]. Therefore, the presence of a gap and insufficient primary stability likely prevented complete osseointegration, despite the use of pure Ti implants in this study.

Nevertheless, direct bone contact was observed on portions of the implant surface in all samples after an adequate healing period, indicating osseointegration, to some extent. Importantly, the experimental Ti alloys demonstrated the same potential for achieving osseointegration as pure Ti.

### 4.4. Bone–Implant Contact

We defined BIC as the percentage of the implant surface in direct contact with bone tissue. BIC values for dental implants in the literature vary widely depending on the surface characteristics. Mechanically polished surfaces (Sa < 0.5 µm) typically show BIC values of 20–40%, acid-etched surfaces (Sa 0.5–1 µm) 40–70%, and sandblasted plus acid-etched surfaces (Sa 1–2 µm) 50–70% [27,35,36,37,38,39]. Generally, increased surface roughness tends to improve BIC. However, excessively rough surfaces (Sa > 2 µm) may promote bacterial adhesion and are, therefore, undesirable [40]. Sa represents the three-dimensional surface roughness parameter, and Ra represents the two-dimensional linear roughness parameter. Although these parameters are not directly equivalent, the surfaces used in the present study (Ra < 0.5 µm) correspond approximately to mechanically polished surfaces. Such surfaces are considered less favorable for achieving high BIC. In addition to this unfavorable surface condition, insufficient primary stability may have contributed to the relatively low BIC observed, even for pure Ti implants. However, as all implants were evaluated under identical experimental conditions, comparisons between materials remained valid. Moreover, the application of surface treatments such as acid etching or sandblasting followed by acid etching could result in higher BIC values.

Both Ti–Ag and Ti–Cu alloys exhibited similar BIC values to those of pure Ti. Although pure Ti is optimal for achieving osseointegration, the experimental alloys did not exhibit inferior performance. Although no significant differences in BIC were observed between pure Ti and Ti–6Al–4V ELI in this study, previous studies have reported lower BIC values and greater connective tissue interposition for Ti–6Al–4V alloys compared to CP-Ti, possibly due to metal ion release [41,42]. In vitro, Al ions released from Ti-6Al-4V alloys have been shown to inhibit osteoblast activity and suppress bone formation [43,44]; Al and V ions may also interfere with hydroxyapatite formation and bone mineralization [45]. The Ti-30Ag and Ti-10Cu alloys investigated in this study may release small amounts of metal ions [18,19]; however, no adverse effects on bone formation were observed. The ion release from these alloys is reportedly lower than that of Ti-6Al-4V alloys [18,19]. Ti-6Al-4V alloys are currently used in dental implants and have a long clinical track record. Herein, the Ti alloys demonstrated bone compatibility comparable to that of Ti-6Al-4V, making them promising candidates for implant materials. Ti–Ag alloys possess anti-biofilm properties as functional dental materials [23]. As current strategies for preventing peri-implantitis primarily rely on plaque control and professional maintenance, Ti–Ag alloys offer an additional material-based approach to preventing biofilm-related infections, making them suitable implant materials.

### 4.5. Limitations

Screw-type implants are currently the dominant design used in clinical practice, whereas cylindrical implants are used less frequently [2]. Although cylindrical implants are still employed in selected clinical situations, their use is more limited than screw-type designs [46,47,48]. Compared to screw-type implants, cylindrical implants cause less surgical trauma to the bone but provide weaker primary stability.

In this study, cylindrical implants were selected because they are easier to fabricate and allow better standardization of experimental conditions. Because the study aimed to investigate the biological responses of bone marrow-contacting metal surfaces, a cylindrical implant design was considered appropriate. However, screw-type implants provide superior primary stability, and differences in hardness and strength among Ti alloys may influence how implant threads engage the bone. Therefore, further studies using screw-type implants are warranted to more accurately evaluate performance under clinical conditions.

After osseointegration, functional loading is applied to the implant via the abutment and superstructure. When the elastic modulus of the implant is higher than that of bone, stress shielding may occur, reducing mechanical stimulation to the surrounding bone and potentially leading to implant loosening. Although low elastic modulus Ti alloys have been developed to mitigate this issue [9,10], sufficient stiffness is also important for narrow and short implants. Previously reported data indicate that the elastic modulus of Ti–Ag alloys is slightly lower (around 105 GPa) than that of pure Ti (approximately 110 GPa), whereas Ti–Cu alloys exhibit slightly higher values (just above 115 GPa) [49]. Further studies are warranted to evaluate long-term mechanical stability under functional loading.

Many studies evaluating the bone compatibility of implants have used X-ray computed tomography (CT). In a preliminary experiment, CT produced severe artifacts around the metal implants. Therefore, CT analysis was not performed. Instead, standardized intraoral radiographic imaging is performed using a positioning device to control the focal distance and film position [24]. This method enabled reproducible radiographs and the comparison of peri-implant bone levels at different observation periods. Combined with histological evaluation and BIC measurements, these analyses provided a sufficient assessment of the bone compatibility of the fabricated implants.

## 5. Conclusions

The experimental Ti–Ag and Ti–Cu alloys demonstrated bone contact comparable to that of pure Ti in a beagle mandibular implantation model. These findings suggest that the developed alloys hold promise as candidate materials for dental implants.

## Data Availability

The original contributions presented in this study are included in the article. Further inquiries can be directed to the corresponding author.

## Abbreviations

The following abbreviations are used in this manuscript:

Al	aluminum
V	vanadium
BIC	bone–implant contact
MMA	methyl methacrylate
H&E	hematoxylin and eosin
CT	computed tomography
